# An Anthropometric-Based Subject-Specific Finite Element Model of the Human Breast for Predicting Large Deformations

**DOI:** 10.3389/fbioe.2015.00201

**Published:** 2015-12-24

**Authors:** Silvia Pianigiani, Leonardo Ruggiero, Bernardo Innocenti

**Affiliations:** ^1^IRCCS, Istituto Ortopedico Galeazzi, Milan, Italy; ^2^BEAMS Department (Bio Electro and Mechanical Systems), École Polytechnique de Bruxelles, Université Libre de Bruxelles, Brussels, Belgium

**Keywords:** breast, finite element method, large deformation, anthropometric, biopsy

## Abstract

The large deformation of the human breast threatens proper nodules tracking when the subject mammograms are used as pre-planning data for biopsy. However, techniques capable of accurately supporting the surgeons during biopsy are missing. Finite element (FE) models are at the basis of currently investigated methodologies to track nodules displacement. Nonetheless, the impact of breast material modeling on the mechanical response of its tissues (e.g., tumors) is not clear. This study proposes a subject-specific FE model of the breast, obtained by anthropometric measurements, to predict breast large deformation. A healthy breast subject-specific FE parametric model was developed and validated by Cranio-caudal (CC) and Medio-Lateral Oblique (MLO) mammograms. The model was successively modified, including nodules, and utilized to investigate the effect of nodules size, typology, and material modeling on nodules shift under the effect of CC, MLO, and gravity loads. Results show that a Mooney–Rivlin material model can estimate healthy breast large deformation. For a pathological breast, under CC compression, the nodules displacement is very close to zero when a linear elastic material model is used. Finally, when nodules are modeled, including tumor material properties, under CC, or MLO or gravity loads, nodules shift shows ~15% average relative difference.

## 1 Introduction

Predicting the mechanical behavior of soft biological tissues is essential for several bioengineering applications. Particularly, estimation of the mechanical behavior of the human breast is fundamental to provide surgeons relevant information to be used in the operation theater. For example, determining the position shift of tumors during biopsy procedures would aid the surgeons to localize and remove cancerous tissues in a more effective way. However, the highly non-linear deformation, to which the breast tissue is subjected to, makes tumors localization, upon gravity or compression loads application, a challenging target (Pathmanathan et al., 2008).

During biopsy, the surgeon must be capable of localizing, precisely and accurately, the tumor that is subjected to shifting its position whether the subject moves with respect to the operation theater coordinate reference system (i.e., absolute reference system). According to the clinical setting, the subject local coordinate reference system changes in function of standing, prone, and supine postures. Each subject postural configuration is strictly related to a specific clinical procedure aiming to perform mammograms, magnetic resonance imaging (MRI), and biopsy, respectively. Frequently used imaging diagnostics techniques, such as two-dimensional (2D) mammograms, are typically performed in Medio-lateral (ML), Medio-Lateral Oblique (MLO), and Cranio-caudal (CC) directions; meanwhile, the subject is oriented in standing position (Kruger et al., 2014). On the contrary, magnetic resonance (MR) images are acquired when the subject is in lying prone position and biopsies are generally performed with the subject in lying supine position (Mertzanidou et al., 2014).

Since 2005 (Carter et al., 2005), substantial progresses have been made in modeling the human breast biomechanics (Han et al., 2012; Mertzanidou et al., 2014). However, the necessity to correlate anatomical features between each local coordinate reference system remains a notably challenging issue, especially in the absence of MRI data providing well-defined geometrical boundaries before and after breast compression or gravity load application. In fact, in order to predict the large deformation of the breast, MRI and computer-aided design (CAD) technologies have been extensively used together with finite element (FE) numerical tools to model the breast mechanical response. Lapuebla-Ferri et al. (2011) and Thanoon et al. (2015) modeled a female breast surgery via finite element analysis (FEA). However, previous works especially aimed to correlate 2D-mammography to three-dimensional (3D) MRI reconstructions, in the attempt of performing non-rigid image registration (Ruiter et al., 2004; Chung et al., 2008; Pathmanathan et al., 2008; Miller and Nielsen, 2010; Hopp et al., 2013; Lee et al., 2013) using FE breast models instead of mere affine transformations (Kruger et al., 2014). In fact, combined FE-affine transformation registration approaches have been recently demonstrated to be more effective than stand-alone image-based registration techniques (Mertzanidou et al., 2014). Pathmanathan et al. (2008) developed a patient-specific FE model of the breast for tumor localization during mammography or biopsy. Nonetheless, for imaging registration studies, Pathmanathan et al. (2008) used MR images for the breast geometrical model reconstruction. The model was used for CC-MLO mammograms matching. They reported that one important limitation of their study was the employment of costly imaging techniques, such as MRI, to reconstruct the subject-specific FE breast model. To the best of the authors’ knowledge, only Chen et al. (2013) proposed a FE-based method to simulate non-linear breast motion by using a 3D scanning system in order to avoid high costs deriving from MRI. The research goal consisted in the development of a FE model enabling the surgeon to predict the breast large deformations upon the effect of compressive loading conditions due to mammography plates compression, avoiding expensive imaging acquisition techniques, such as MRI, or complicated customized set-ups, such as photogrammetry systems or body scanners (Chen et al., 2013). The main unquestionable advantages of using MRI devices and body scanners is the possibility of obtaining an accurate discretization of the breast geometric features due to their high resolution and the potentiality to include internal structures, such as fibroglandular or tumorous tissues, so generating an “exact” reconstruction of all anatomical structures and consequently breast geometrical boundary conditions. However, these advantages are counterbalanced by some disadvantages of fundamental importance for clinical applications, such as biopsy procedures. The disadvantage of performing MRI scans still remains, as already mentioned, the high cost, rather than the availability of MRI devices for dedicated subject-specific data acquisitions. MRI devices are usually employed also for other clinical purposes and, due to their high cost, they generally serve more than one hospital department or healthcare district or hospital. Additionally, for both MRI and body scanners data acquisition systems, the model development phases (e.g., data acquisition, 2D MR images segmentation, and 3D reconstruction) are time expensive and need qualified engineering expertise, rather than being unusual to common clinical rolling stock, such as in the case of body scanners. Up to date, mammography is the most cost-effective imaging diagnostic tool utilized for breast cancer detection. In fact, the latter technique is not only cost-effective but also the only technique capable of detecting micro-calcifications. 2D-mammograms are always available to the surgeons for preventive screening, pre-planning surgery, and patients’ follow-ups.

Material characterization studies found the fibroglandular tissue being stiffer than the fatty tissue and cancerous tissue being stiffer than the fibroglandular one (Carter et al., 2005). Consequently, it is hypothesized that tumors are displaced from their initial position when the coordinates of the subject local reference system change. Essentially, the subject’s tumor initial position changes as function of large breast deformations. Therefore, the tumor spatial coordinates change with respect to the local coordinate system if the subject is oriented in a standing, supine, or prone position (Pathmanathan et al., 2008). The relative shift of the tumor with respect to the local coordinate system, due to breast large deformations, prevents the surgeons to track its position and efficiently remove it by biopsy.

Breast tumors change their position according to the evolution stage and their material properties are different from other breast tissues. The ductal carcinoma *in situ* (DCIS) rises at early stage. It is present in the milk duct without affecting the surrounding breast tissue (Wang et al., 2015) and it is non-life-threatening, but it can increase the risk of having an invasive breast cancer (Pape-Zambito et al., 2014) and it is mainly diagnosed by mammography. The invasive ductal carcinoma (IDC) is a tumor that begins to invade the ducts surrounding tissues, such as the glandular tissue, overtime evolution of a DCIS nodule. As a matter of fact, to the best of the authors’ knowledge, even if different material properties have been identified for breast tumorous and fibroglandular tissues, there is not yet clear understanding about the most suitable material modeling design approach to be adopted for breast large deformations prediction. For example, previous studies (Ruggiero et al., 2014) highlighted substantial differences (i.e., from 20% to more than 80%) in approximating non-linear models with their linear or pseudo-non-linear formulation. In this context, a parametric study considering the presence of fibroglandular and tumorous tissues has been also carried out to estimate the influence of nodules typology, size, and location, considering a subject-specific geometry.

Therefore, the proposed study aims (i) to validate the developed customized breast model based on anthropometric measurements, via 2D-mammograms and (ii) to analyze the tumors shift in function of linear and non-linear constitutive material modeling and breast loading conditions. On the contrary of other studies such as the ones reported by Carter et al. (2005) and the one, for example, conducted by Tanner et al. (2006), the development of an image-guided approach is not the goal of the presented work.

## 2 Materials and Methods

This study proposes the use of a customized breast model based on anthropometric measurements to predict the breast large deformation due to compressive loading conditions achieved during mammography. Furthermore, the developed and validated model was used to study the effect of nodules size and position together with material modeling assumptions, i.e., linear or non-linear, on the breast biomechanics. Conversely to other studies (Tanner et al., 2006), the model validation has not been performed for nodules tracking. The main reason lies in the fact that the analyzed constitutive models have not been used in combination with imaging techniques, such as MRI. In fact, other studies (Tanner et al., 2006) derived surface displacement boundary conditions from a 3D non-rigid image registration using MRI. In this study, the FE model boundary conditions do not change adaptively according to quantitative information (i.e., breast substructures contours) extracted from MRI data.

### 2.1 Customized Breast Model

Primary requirement for performing anthropometric measurements is the individuation of reference fiduciary anatomical markers (Brown et al., 1999). In this study, an anthropometric measurement methodology, based on the one introduced by Brown et al. (1999), was utilized to create a reference volumetric grid, defining a 3D space where the coordinates of measurement points constitute B-splines control points coordinates. The B-splines approximate the subject-specific breast curvature, generating a parametric customized model of the breast.

**Figure 1:**
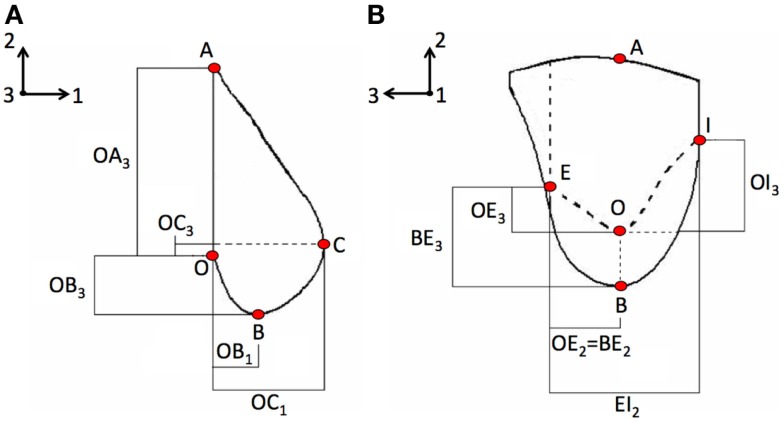
**Anthropometric measurements were based on the methodology adopted by Brown et al. (1999) and consisted in measuring the relative distances among fiduciary breast markers on the (A) coronal plane and on the (B) sagittal plane**. Directions 1, 2, and 3 correspond to the AP, CC, and ML directions, respectively. Letters indicate the control points used for building the parametric model, e.g., A is the attachment point to the pectoralis major muscle, B is the base of the breast, and C the nipple.

Six reference fiduciary breast markers were identified and relative distances among them were measured by a ruler. Upon ethical approval, measurements were taken multiple times on six subjects in standing position, under gravity load conditions, in order to verify the measurement reliability and the accuracy error. The identified markers position and the measured relative distances are depicted in Figure 1. The fiduciary markers corresponded to the B-spline control points. Once the reliability was proved, the parametric model was used to generate a patient-specific breast reconstruction of 1 patient based on 10 known parameters, corresponding to the relative distances measured among the fiduciary markers, i.e., B-spline control points relative distances.

### 2.2 Case Studies

The effect of loading on breast large deformation was investigated for a healthy and a pathological breast, i.e., embedding tumorous tissues as nodules inclusions. The internal tissues of the breast, for both healthy and pathological case studies, were modeled as mere partitions of the breast geometry. No imaging techniques were used to define the geometrical boundaries of the different tissues composing the breast. Precisely, the parametric study took into account the following case studies, representing various loading conditions: (i) CC mammographic compression, (ii) 45° MLO mammographic compression, and (iii) gravity effect in supine lying position.

**Figure 2:**
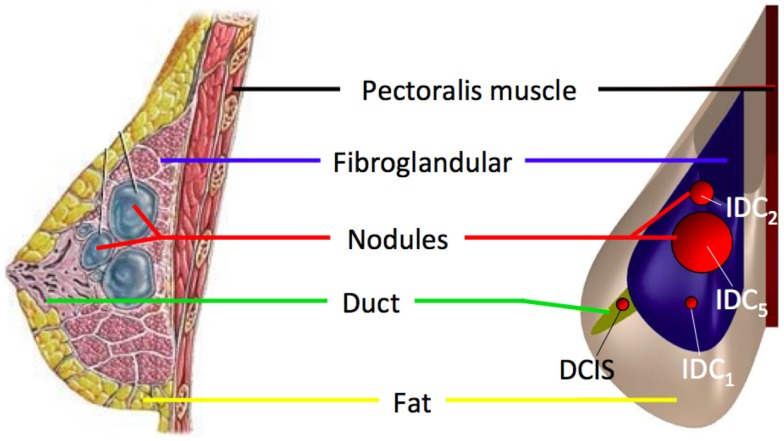
**The healthy breast parametric FE model was modified by adding spherical inclusions (red) mimicking the presence of IDC and DCIS nodules**. IDC nodules were of 10, 20, and 50 mm diameter.

The pathological breast model was assumed to be characterized by the inclusion of solid spherical geometrical partitions (Figure 2), with their superficial nodes tied to the surrounding elements, representation of two tumors typologies: DCIS and IDC.

For each case study, linear elastic, neo-Hookean, and Mooney–Rivlin hyperelastic material models were considered (Table 1). The predicted breast deformation in the case of CC compression of a healthy breast was utilized for validation, comparing the subject mammogram to the output of the subject-specific parametric FE model. The impact of IDC nodules size was also evaluated taking into account nodules dimensions of 10, 20, and 50 mm diameter, during CC compression. Nodules material modeling was also evaluated, under CC compression, tracking the nodules displacement when a linear elastic or a hyperelastic Mooney–Rivlin material model is used. Finally, nodules shift was taken into account when DCIS or IDC nodules are coexistent within the same breast under CC and MLO compression and gravity load. In this last case study, the nodules displacement was analyzed in function of the Mooney–Rivlin material parameters. For all simulations, two cases were compared: (i) nodules with assigned tumorous material parameters and (ii) nodules characterized by the same material parameters of the surrounding fibroglandular tissue. The latter was used as reference model to evaluate the incidence of tumorous material properties on nodules displacement.

**Table 1:** **Material properties for all linear and non-linear constitutive models used in the study**.

Component	Material model	Material parameters			Reference
		E (kPa)	*c*_10_ (kPa)	*c*_01_ (kPa)	
Fat	Linear elastic	7.3	–	–	Sarvazyan et al. (1994) and Krouskop et al. (1998)
					Wellman et al. (1999) and Samani and Plewes (2004)
	neo-Hookean	–	3	–	del Palomar et al. (2008)
	Mooney–Rivlin	19.8^a^	2	1.3	Yin et al. (2004)
Fibroglandular	Linear elastic	42.7	–	–	Sarvazyan et al. (1994) and Krouskop et al. (1998)
					Wellman et al. (1999) and Samani and Plewes (2004)
	neo-Hookean	–	12	–	del Palomar et al. (2008)
	Mooney–Rivlin	34.8^a^	3.5	2.3	Yin et al. (2004)
Tumor	Linear elastic	912.39	–	–	Sarvazyan et al. (1994) and Krouskop et al. (1998)
					Wellman et al. (1999) and Samani and Plewes (2004)
	neo-Hookean	–	–	–	del Palomar et al. (2008)
	Mooney–Rivlin	99.6^a^	10	6.6	Yin et al. (2004)
Muscle	Linear elastic	110	–	–	Babarenda Gamage et al. (2012)
Plates	Linear elastic	2,400	–	–	–

*^a^Approximated value (MRA) calculated from equation (3)*.

### 2.3 Finite Element Model

The subject-specific breast FE model was characterized by linear tetrahedral elements (Abaqus v6.12, Dassault Systmes, Waltham, MA, USA). A dynamic explicit analysis was performed to simulate a quasi-static compression of the breast. In fact, as also reported by previous studies, an implicit analysis might be computationally expensive for such complicated numerical problem (Carter et al., 2005). When using an implicit solver, higher computation times are necessary especially when, such as in this study, contact boundary conditions and large deformations are present (Han et al., 2012). However, quasi-static loading conditions can be simulated using a dynamic explicit solver as long as inertial forces are negligible.

#### 2.3.1 Loading and Boundary Conditions

**Figure 3:**
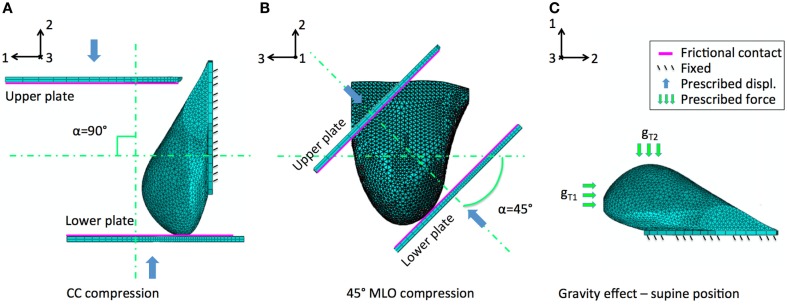
**Breast large deformations occur upon mammography or subject posture changes**. The developed model was analyzed considering the effect of **(A)** CC mammographic plates compression, **(B)** 45° MLO compression, and **(C)** gravity load. The latter intervening during subject positioning during biopsy. Main displacements directions 1, 2, and 3 correspond to the AP, CC, and ML directions, respectively.

**Figure 4:**
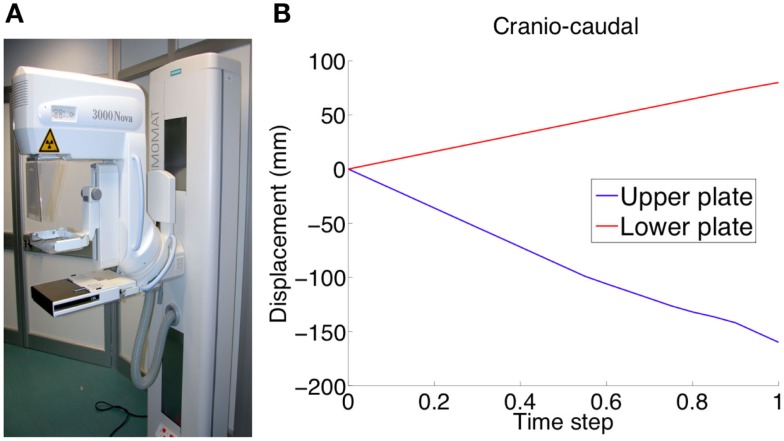
**Experimental validation was performed using mammographic data acquisitions (A)**. The plates move relatively to each other in order to compress the breast for optimizing the x-ray irradiation. The applied plate displacements during CC compression (U_2_ direction) is depicted in **(B)**.

The three main analyzed case studies correspond to three loading conditions, two related to mammography loading and one to gravity effect. Both CC and 45° MLO breast compression were simulated referring to real mammograms set-ups (Figures 3A,B). As already highlighted by previous studies, non-physical reaction forces can be generated if the nodal displacements are imposed explicitly (Lee et al., 2013), consequently, mammographic compression plates were modeled. The plates displacement was imposed according to the one measured during mammography (Figure 4). The mesh nodes in correspondence of the pectoralis muscle were considered fixed. The remaining nodes were instead characterized by all six degrees of freedom. Since breast-plates friction values range between 0 and 1 (Yin et al., 2004; Shih et al., 2010; Hsu et al., 2011), a sliding contact with friction coefficient of 0.5 was imposed between plates and breast surface. The third loading condition was a gravity load. Considering that the customized breast model was obtained with the subject in standing position, a two-step analysis was implemented to simulate the subject local coordinate reference system rotation, leading the subject to the lying supine position from the standing one. The first step consisted in the application of an “anti-gravity” distributed load, erasing the gravity effect due to the standing position. Successively, gravity was reapplied by the second step, considering the gravity force perpendicular to the subject coronal plane (Figure 3C).

#### 2.3.2 Material Modeling

The difficulties in modeling the breast mechanical behavior are majorly due to its large deformation (Azar et al., 2002; Ruggiero et al., 2014). For this reason, the adopted material model can play a key role for the estimated breast mechanical response when subjected to different loading conditions. In order to individuate the most suitable material model, the performances of three constitutive laws were analyzed. The considered material models have been linear elastic, Mooney–Rivlin, and neo-Hookean. For all material models, incompressibility was assumed. Material properties for the linear elastic model were averaged from the ones proposed in literature by Sarvazyan et al. (1994), Krouskop et al. (1998), Wellman et al. (1999), and Samani and Plewes (2004), whereas Mooney–Rivlin and neo-Hookean coefficients were taken from Yin et al. (2004) and del Palomar et al. (2008), respectively. The Mooney–Rivlin and neo-Hookean models were derived from the generalized Rivlin model:

(1) $$\Psi =\sum_{p,q=0}^{N}\;c_{\text{pq}}{\left({\bar{I}}_{1}-3\right)}^{p}{\left({\bar{I}}_{2}-3\right)}^{q}+\sum_{m=1}^{M}\;D_{m}{\left(J-1\right)}^{2m}$$

where Ψ is the strain energy density function, ${\bar{I}}_{1}$ and ${\bar{I}}_{2}$ are the first and the second invariant of the deviatoric component of the left-Cauchy–Green deformation tensor **F**, *J* = *det*(**F**), *c_pq_* are material constants related to the distortional mechanical response, and *D_m_* are material constants related to the volumetric response. For an incompressible Mooney–Rivlin material model where *c*_00_ = 0, *N* = 1, *M* = 1, and *D*_1_ = 0, equation (1) can be written as:

(2) $$\Psi =c_{10}\left({\bar{I}}_{1}-3\right)+c_{01}\left({\bar{I}}_{2}-3\right)$$

The Mooney–Rivlin material model parameters can be related to the Young’s modulus of its linear elastic approximation by:

(3) $$E=6\left(c_{10}+c_{01}\right)$$

For an incompressible neo-Hookean material model, supposing *c*_01_ = 0, equation (2) can be rewritten as:

(4) $$\Psi =c_{10}\left({\bar{I}}_{1}-3\right).$$

The CC compression case study was used to select the most suitable constitutive law and validate the results comparing the estimated breast deformation to the ones obtained during mammography. Material properties of each material model analyzed for fat, fibroglandular, and tumorous tissues are listed in Table 1. Plates were modeled as linear elastic and characterized by polyvinyl chloride (PVC) material properties.

### 2.4 Validation

Mammograms were obtained using a MAMMOMAT 3000 Nova (Siemens AG, Erlangen, Germany). The estimated deformations of the breast under CC and 45° MLO compression, obtained adopting linear and non-linear formulations, were validated comparing the FEA predictions to the CC and MLO mammograms, respectively. The projected images, depicting the compressed breast contour was superimposed to the FE deformed breast model contour. In order to quantitatively estimate the images superimposition, the overlapping area between mammograms and calculated deformation was determined and compared. Overlappings were expressed in percentages. Consequently, a value of 100% was considered when the areas were fully superimposed (i.e., 100% accuracy). Upon validation, the selected material model was used to carry out a parametric study aiming to evaluate the influence of tumors displacements in function of nodules typology, size, and location.

## 3 Results

**Figure 5:**
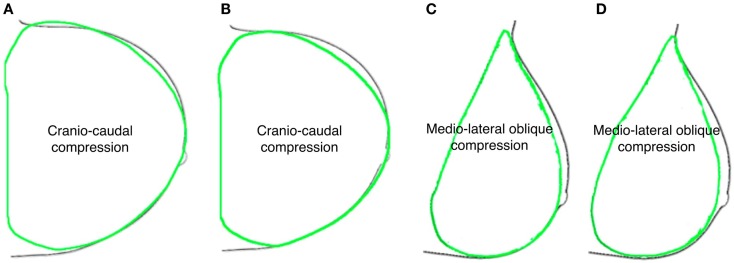
**Predicted deformed breast contours (green) were superimposed to mammograms ones (black) obtained assuming healthy breast (A–C) neo-Hookean and (B–D) Mooney–Rivlin material properties, for CC and MLO compression, respectively**.

The reported results are related to one of the six subjects used for the anthropometric measurements. While repeated measurements on six subjects were performed to determine the accuracy of the fiduciary markers position, only one subject was used for the FE model generation. For all performed dynamic explicit FE simulations, the ratio of the kinetic energy to the internal energy showed to be below 5%, thus quasi-static conditions are assumed due to the negligible influence of the dynamic effect (Han et al., 2012).Considering the healthy subject clinical condition, i.e., absence of nodules, the linear elastic modeling approach provides unrealistic deformation predictions, while the Mooney–Rivlin and neo-Hookean material models show to be able to estimate breast-plates compression with the same accuracy. The computation time is equivalent when modeling a healthy breast adopting either a neo-Hookean or a Mooney–Rivlin material model. Simulating a pathological breast, including the presence of nodules, the computation time difference almost doubles utilizing a neo-Hookean constitutive law. The subject-specific breast model deformation was validated by superimposing its deformed shape to the subject CC and MLO mammogram contours (Figure 5). For the healthy breast case study, i.e., absence of nodules, the prediction accuracy was calculated. The predicted deformed shapes were superimposed to mammograms and the overlapping and non-overlapping areas were calculated. The overlapping areas values, after CC compression, are ~97% for the Mooney–Rivlin material model and ~98% for the neo-Hookean material model. Applying a MLO compression, the calculated overlapping are ~91 and ~94% for the Mooney–Rivlin and for the neo-Hookean material model, respectively. The computation time is 1.5 h for models embedding a Mooney–Rivlin and 2.5 h for a neo-Hookean constitutive law, using a dual-core 1.7 GHz Intel Core i7 8 GB RAM. The impact of nodules linear elastic vs. hyperelastic Mooney–Rivlin material modeling was evaluated tracking the displacement of the nodes within the nodules geometrical partitions. The nodules displacement was calculated as the average displacement of the nodes belonging to the nodules geometrical partition.

### 3.1 Effect of Nodules Size and Material Modeling

**Figure 6:**
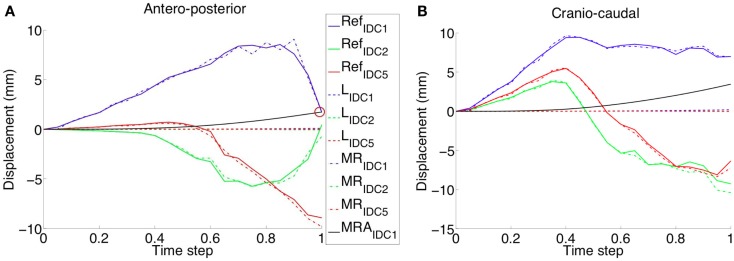
**The validated healthy breast model was modified adding nodules of different size and CC compression was applied**. The reference displacement trends (e.g., Ref_IDC1_ for an IDC nodule of 10 mm diameter) are related to the same nodes, belonging to the nodules partitions, but characterized by fibroglandular material parameters. Displacements relative differences, between nodules modeled with linear and non-linear material properties, are reported along the **(A)** AP and **(B)** CC directions. Linear elastic material modeling of the nodules (e.g., L_IDC1_ for an IDC nodule of 10 mm diameter) results in a very small displacement for all the evaluated nodule sizes. On the contrary, Mooney–Rivlin material modeling gives in output definitively higher displacements along both AP and CC directions. A linear approximation (i.e., MRA_IDC1_ for an IDC nodule of 10 mm diameter) of the Mooney–Rivlin material parameters, characterizing the tumor, approximates the nodule shift final value (red circle), modeled using the Mooney–Rivlin material model, only in the AP direction.

Under CC compression (Figure 6), along both Antero-posterior (AP) and CC directions (Figure 6A), when a Mooney–Rivlin model is used, the nodules final displacement is 8.74–520 times higher than the final displacement predicted adopting a linear elastic model. During CC compression, the higher discrepancy is present along the CC direction, in the case of the IDC_5_ nodule. Along the AP direction, the nodules displacement is inversely proportional to the nodule size in the linear elastic case; instead, no correlation between nodules displacement and size can be made in the non-linear case. In fact, for example, IDC_2_ moves −0.711 mm along AP direction and −10.39 mm along CC direction, whereas IDC_3_ moves −9.84 mm along AP direction and −7.815 mm along CC direction.

### 3.2 Effect of Nodules Material Properties and Prescribed Load

**Figure 7:**
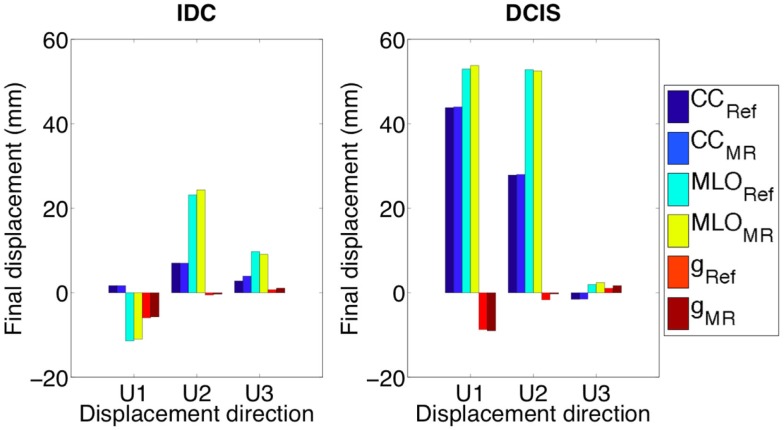
**Both IDC and DCIS nodules final displacements are very close to the displacements of the same geometrical partitions when characterized by Mooney–Rivlin material properties related to the fibroglandular tissue (e.g., CC_Ref_)**. U1, U2, and U3 are AP, CC, and ML directions, respectively.

Under the effect of CC compression, modeling IDC_1_ as linear elastic material model, direct approximation of the Mooney–Rivlin material model (Ruggiero et al., 2014) according to the relation equation (3), the final displacement along the AP direction is comparable with one estimated adopting the non-linear approach (Figure 6A). However, the displacement trends and the final displacement along the CC direction are notably different. Surprisingly, under CC and MLO compression and gravity load, no appreciable difference is present when modeling the nodules as tumorous or fibroglandular tissues (Figure 7). Changing the prescribed loading conditions, results show that the DCIS nodule moves more than the IDC one. In fact, the maximum final IDC and DCIS displacements are ~24 and ~54 mm along CC direction for IDC and both CC and AP directions for DCIS, respectively. However, for both IDC and DCIS nodules, the maximum final displacement relative difference between tumorous and reference case is around only 1.4 mm.

## 4 Discussion

First aim of this study is to develop and validate a subject-specific model capable of predicting the healthy breast deformation during compression. Anthropometric measurements robustness was tested on six healthy subjects performing repeated measurements. A maximum accuracy error of 8 mm is determined measuring the spatial relative distances among the parametric model control points (Figure 1). The error may be mainly attributed to the measurement instrument (i.e., a ruler was used), and to the parallax error, operator dependent. The measurement error is considered to be very difficult to minimize with the adopted non-invasive and low-cost methodology. However, due to the fact that the prescribed plate displacements for CC compression was 160 and 80 mm for top and bottom plate, respectively, the anthropometric measurement error can be considered negligible. The CC and MLO mammograms validate the predicted deformation. Images superimposition demonstrates that no relevant difference is present if a neo-Hookean or a Mooney–Rivlin material model is adopted (Figure 5). Modeling the healthy breast, the contour matching evaluation between numerical predictions and mammograms highlight higher accuracy for the neo-Hookean material model. The overlapping difference between mammograms and predicted breast deformation is 1% in CC compression and 3% in MLO compression. The highest accuracy of the neo-Hookean material model is also reported by Mertzanidou et al. (2014). However, the perspective of using the proposed methodology in a clinical setting justified the choice of a cost-effective modeling approach. Consequently, the Mooney–Rivlin model was selected to perform a further parametric study aiming to verify the impact of nodules material modeling on their shift due to three loading conditions. Precisely, prescribed loads were mammographic CC and MLO compression, and gravity load. Results show that modeling the nodules employing a linear elastic constitutive law does not provide any information about nodules displacement, under CC compression. The nodules displacement predicted by the developed FE model, when nodules are modeled as linear elastic, is lying in between 0.015 and 0.185 mm. To the best of authors’ knowledge, no reliable technique is available to track the nodules displacement; consequently, no range of motion is available as reference to validate the reported results. However, modeling the nodules as non-linear Mooney–Rivlin provides appreciable displacements with respect to the linear elastic case study. As reported by equation (3), the adopted Mooney–Rivlin parameters for fat, fibroglandular, and tumorous tissues (Yin et al., 2004) can be approximated to a unique coefficient corresponding to the Young’s modulus of an incompressible linear elastic model. The calculated linear elastic approximations are ~20, ~35, and ~100 kPa, respectively, for fat, fibroglandular, and tumorous tissues. In light of these approximations, unmatching predictions could be due to the fact that, according to the average material parameters determined from the literature (Sarvazyan et al., 1994; Krouskop et al., 1998; Wellman et al., 1999; Samani and Plewes, 2004), the nodules average Young’s modulus is ~900 kPa for an IDC tumor (~200 kPa for a DCIS), basically an order of magnitude more of the linear approximation obtained from Mooney–Rivlin material characterization. Consequently, the deformation of the nodes belonging to the nodules geometrical partitions are expected to be lower because of higher stiffness values. On the other hand, the non-linear Mooney–Rivlin material properties provide definitively lower nodules stiffness and therefore higher displacement predictions. Approximating the Mooney–Rivlin model with its linear elastic formulation confirms that the stiffness relative difference, among the breast tissues components, is responsible for the higher nodules displacement values (Figure 6B). However, the relative stiffness difference between fibroglandular and tumorous tissues (Figure 7), i.e., E_MRA(tum)_ = 2 E_MRA(gland)_, does not really impact on the nodules displacement when they are modeled as non-linear. Maximum relative differences between nodule modeling and reference case study for the IDC nodule are 30% in CC compression (U_3_ direction), 7% in MLO compression (U_3_ direction), and 55% under the effect of gravity load (U_2_ direction). For the DCIS nodule, maximum relative differences are 3% in CC compression, 20% in MLO compression, and 500% under gravity load. In other words, for example, considering the DCIS nodule, out of the maximum relative difference of ~1.4 mm with respect to the reference model, an absolute maximum nodule displacement of ~0.28 mm (i.e., gravity load) implies a relevant nodule shift under gravity effect. On the contrary, considering the mammographic plates compression, a nodule shift of ~0.15 and ~0.85 mm for CC and MLO, respectively, is calculated along the CC direction, whereas the absolute nodule displacement is ~44 and ~54 mm for CC and MLO, respectively. The average relative differences with respect to the reference are 15.5 ± 19.39% with *p*-value <0.05 (*n* = 9) (where *n* is the number of observations, corresponding to nine displacements: three loading conditions per three directions) only for IDC while they are equal to 62.7 ± 163.81% with *p*-value >0.05 (*n* = 9) for DCIS. Therefore, considering an average relative difference of 15.5% for the IDC nodule, it can be stated that the nodule material modeling does not really impact on the nodule displacement, while nothing can be said for the DCIS nodule. The study presents two limitations to take into account in the interpretation of the results. The first limitation consists in the use of only one subject to build the parametric breast model. The second limitation is the absence of an experimental set-up validating the nodules displacement prediction. However, the validation of nodules shift was not fundamental for the goal of the study. The primary goal in evaluating nodules shift is the effect of nodules material properties and models on their displacement.

## 5 Conclusion

In conclusion, the presented study introduced a validated patient-specific FE model based on anthropometric measurements demonstrating that the healthy breast large deformations can be predicted during CC and MLO mammographic compression. In spite of the mentioned limitations, results show that current tumorous tissue material characterization is insufficient to appreciate nodules shift during breast compression also when non-linear material modeling is adopted. Future works must incorporate robust material characterization for breast fatty, fibroglandular, and tumorous tissues, using different non-linear constitutive material models, in order to provide more exhaustive considerations about nodules shift modeling. Furthermore, a higher number of subjects should be considered to state more general conclusions. Further research must be addressed in order to evaluate more exhaustively the influence of breast tissue material models on their biomechanics.

## Conflict of Interest Statement

The authors declare that the research was conducted in the absence of any commercial or financial relationships that could be construed as a potential conflict of interest.
